# Growth, condition, and maturity schedules of an estuarine fish species change in estuaries following increased hypoxia due to climate change

**DOI:** 10.1002/ece3.4236

**Published:** 2018-06-25

**Authors:** Alan Cottingham, Peisheng Huang, Matthew R. Hipsey, Norman G. Hall, Eloise Ashworth, Joel Williams, Ian C. Potter

**Affiliations:** ^1^ Centre for Sustainable Aquatic Ecosystems Harry Butler Institute School of Veterinary and Life Sciences Murdoch University Murdoch WA Australia; ^2^ UWA School of Agriculture and Environment The University of Western Australia Crawley WA Australia; ^3^ Department of Primary Industries and Regional Development Western Australian Fisheries and Marine Research Laboratories Hillarys WA Australia

**Keywords:** *Acanthopagrus butcheri*, biological plasticity, climate change, environmental indicator, freshwater discharge, hypoxia, microtidal estuaries, Sparidae

## Abstract

Understanding challenges posed by climate change to estuaries and their faunas remains a high priority for managing these systems and their communities. Freshwater discharge into a range of estuary types in south‐western Australia between 1990 and 2015 is shown to be related to rainfall. This largely accounts for decreases in discharge in this microtidal region being more pronounced on the west coast than south coast, where rainfall decline was less. Results of an oxygen‐balance model imply that, as demonstrated by empirical data for the Swan River Estuary, declines in discharge into a range of estuary types would be accompanied by increases in the extent of hypoxia. In 2013–15, growth and body condition of the teleost *Acanthopagrus butcheri* varied markedly among three permanently open, one intermittently‐open, one seasonally‐closed and one normally‐closed estuary, with average time taken by females to reach the minimum legal length (MLL) of 250 mm ranging from 3.6 to 17.7 years. It is proposed that, in a given restricted period, these inter‐estuary variations in biological characteristics are related more to differences in factors, such as food resources and density, than to temperature and salinity. The biological characteristics of *A. butcheri* in the four estuaries, for which there are historical data, changed markedly between 1993–96 and 2013–15. Growth of both sexes, and also body condition in all but the normally‐closed estuary, declined, with females taking between 1.7 and 2.9 times longer to attain the MLL. Irrespective of period, body condition, and growth are positively related. Age at maturity typically increased between periods, but length at maturity declined only in the estuary in which growth was greatest. The plasticity of the biological characteristics of *A. butcheri*, allied with confinement to its natal estuary and ability to tolerate a wide range of environmental conditions, makes this sparid and comparable species excellent subjects for assessing estuarine “health.”

## 1 INTRODUCTION

Estuaries are among the most productive of aquatic ecosystems and thus provide an abundant source of food for fishes and other fauna (Blaber & Blaber, 1980; Elliott & Whitfield, 2011; Whittaker & Likens, 1975). They are also widely used for recreational purposes such as fishing and boating. During recent decades, the hydrological characteristics of these important systems (and coastal seas) have become modified in many parts of the world as a result of changes in rainfall and other effects of climate change (Altieri & Gedan, 2015; Hewitt, Ellis, & Thrush, 2016). For example, marked reductions in precipitation have led to declines in discharge and thus the effectiveness of flushing (Altieri & Gedan, 2015; Tweedley, Warwick, & Potter, 2016). Such declines, in turn, increase residence time and thus the retention of nutrients and organic material and, consequently, increases in primary productivity, which can ultimately lead to marked eutrophication and the development of oxygen‐depleted “dead” zones (Diaz & Rosenberg, 2008; Thronson & Quigg, 2008). This problem is increasing in estuaries throughout the world (Paerl, Hall, Peierls, & Rossignol, 2014). As estuaries are often surrounded by areas of agricultural, industrial and urban developments and therefore frequently receive substantial amounts of nutrients and organic material through runoff from surrounding land and by direct input, these systems are particularly prone to developing these detrimental effects. The effects of climate change on the discharges into estuaries, and thus of their flushing and the prevalence of oxygen‐depleted dead zones, have sometimes been exacerbated by the construction of structures, such as dams and reservoirs, for diverting river flow for agriculture, industry and/or human water usage (Mehran et al., 2017; Stewardson et al., 2017).

Studies in North America have shown that hypoxia in estuaries can lead to changes in the biological characteristics of certain fish species within those systems (e.g., Breitburg, Hondorp, Davias, & Diaz, 2009; Eby et al., 2005; Wu, 2002). For example, when some species are exposed to hypoxia, they often aggregate in better‐oxygenated environments, which can lead to inhibitory density‐dependent effects (Campbell & Rice, 2014; Eby & Crowder, 2002; Tuckey & Fabrizio, 2016). Such fish species thus provide excellent, albeit under‐used, test subjects for elucidating the degree and direction of shifts in the environmental quality of aquatic ecosystems (Izzo et al., 2016).

The characteristics of estuaries in microtidal regions make these systems particularly susceptible to accumulating nutrients and organic material and thus to the development of hypoxia (Tweedley, Warwick et al., 2016). Thus, in microtidal regions with Mediterranean climates, such as south‐western Australia, one of the 25 regions worldwide recognized as global biodiversity hotspots by Myers, Mittermeier, Mittermeier, Da Fonseca, and Kent (2000), the flushing effect of tides in estuaries is far less than in macrotidal systems. As rainfall in south‐western Australia occurs predominantly in winter and early spring, nutrients and organic material are mainly flushed from the estuary during that period. Consequently, any decrease in winter flushing increases the likelihood of oxygen‐depleted zones forming in the following summer when flushing is least and temperatures are greatest and conditions for microbial utilization of nutrients and organic material are thus optimal (Cottingham, Hall, & Potter, 2016; Hodgkin & Hesp, 1998; Spencer, 1956; Tweedley, Warwick, Clarke, & Potter, 2014).

The possession by the estuaries of south‐western Australia of typically a narrow entrance channel (lower estuary) that leads to a wide central basin area (middle estuary) results in attenuation of the already relatively weak tidal action (Spencer, 1956). This, in turn, means that tidal influence is particularly small in the saline lower reaches of the rivers (upper estuary) that discharge into the basins. It thus follows that the flushing of south‐western Australian estuaries, and of microtidal estuaries elsewhere, is largely governed by freshwater discharge. Furthermore, the extent to which the estuaries of south‐western Australia are connected with the sea varies, which will clearly affect the extent to which these systems are flushed. Thus, although a number of estuaries in this region remain permanently open, for example, the Swan River Estuary and Walpole‐Nornalup Inlet, sand bars form at the mouths of many others. Such bars close the estuary from the ocean either intermittently during the year, for example, Moore River Estuary, seasonally, for example, Wilson Inlet, or for a number of years, for example, Wellstead Estuary, which is therefore termed a normally‐closed estuary (Hodgkin & Hesp, 1998; Potter, Hyndes, & Baronie, 1993).

South‐western Australia has experienced one of the greatest declines in rainfall of any region in Australia during the last century (Australian Bureau of Meteorology, 2018a), which has reduced runoff and thus discharge into estuaries (Petrone, Hughes, Van Niel, & Silberstein, 2010; Silberstein et al., 2012; Smith & Power, 2014). Between 1975 and 2004, the ~15% decline in rainfall in the Perth region, on the west coast of this region, was accompanied by a reduction of over 50% in the runoff into reservoirs (Bates, Hope, Ryan, Smith, & Charles, 2008). The trend map produced by the Australian Bureau of Meteorology (2018a) shows, however, that the extent to which rainfall has declined since 1970 becomes progressively less eastward from the lower west coast along the south coast, with changes becoming minimal in the region of the Wellstead Estuary, the most eastern of the estuaries that are the focus of this study. Still further east, rainfall even increased slightly during approximately the same period in the catchment of one estuary but did not change significantly in the catchments of two other estuaries (Hoeksema, Chuwen, Tweedley, & Potter, 2018).

On the basis of data collected in 1995–2010, the extent of hypoxia in the bottom waters of the Swan River Estuary on the lower west coast of Australia is inversely related to freshwater discharge, which declined during those years (Cottingham, Hesp, Hall, Hipsey, & Potter, 2014). The degree to which the Swan River Estuary has become degraded is emphasized by the conclusion of Cloern, Foster, and Kleckner (2014) that it was the second most eutrophic of the 131 estuarine‐coastal ecosystems from around the world for which they had collated data. Although oxygen concentrations in bottom waters of south‐western Australian estuaries have been continuously monitored only in the Swan River Estuary, the limited data available for the Moore River and Blackwood River estuaries indicate that the prevalence of hypoxia in the bottom waters of these systems has likewise increased following declines in flow (Anderson, 2004; Brearley, 2013).

The increase in the extent of bottom water hypoxia in the Swan River Estuary since the 1990s was accompanied in the demersal Black Bream *Acanthopagrus butcheri* by reductions in growth and body condition and a strong tendency for aggregating in shallow, better‐oxygenated areas, which led to a marked increase in density in those waters (Cottingham, Hall, Hesp, & Potter, 2018; Cottingham et al., 2014). These trends parallel those recorded for fish species in North America when exposed to hypoxia (e.g., Campbell & Rice, 2014; Eby & Crowder, 2002). In the Swan River Estuary, the increased prevalence of hypoxia was also accompanied by a decrease in length at maturity and increase in age at maturity of *A. butcheri*, which is consistent with life‐history theory (Stearns & Koella, 1986). The changes in the biological characteristics of *A. butcheri* in the Swan River Estuary with marked reductions in oxygen concentrations occurred either directly through effects on metabolism or indirectly through, for example, density‐dependent mechanisms or changes in prey composition (Cottingham et al., 2014).

The plasticity of the biological characteristics of *A. butcheri* is reflected in marked differences between its growth and maturity schedules among south‐western Australian estuaries in which the physico‐chemical, hydrological and morphological characteristics and composition of potential food sources differ markedly (Brearley, 2005; Chuwen, Platell, & Potter, 2007; Gardner et al., 2013; Sarre, Platell, & Potter, 2000; Sarre & Potter, 2000). Variations in the growth of *A. butcheri* have sometimes been positively related to temperature and sometimes inversely (Cottingham et al., 2018; Doubleday et al., 2015) and the particularly slow growth rate in the Moore River Estuary in south‐western Australia was considered to be related inter alia to the very low salinities that characterize this system (Sarre & Potter, 2000).

As *A. butcheri* is relatively long‐lived (up to 30 years) and typically restricted to its natal estuary for the whole of its life cycle (Morison, Coutin, & Robertson, 1998; Potter et al., 2008; Sarre & Potter, 2000; Williams, Jenkins, Hindell, & Swearer, 2013), it is exposed, throughout life, to any ongoing deleterious changes in environmental quality within the estuary (Cottingham et al., 2016, 2018). This sparid, which is the most important recreational fish species in southern Australian estuaries (Gomon, Bray, & Kuiter, 2008; Jenkins, Conron, & Morison, 2010; Kailola et al., 1993; Lenanton & Potter, 1987), thus constitutes an ideal test subject for exploring the ways and extents to which this and comparable species can respond to the effects of climate change and act as an indicator of “health” of estuaries (Bortone, Martignette, & Spinelli, 2006; Valesini, Cottingham, Hallett, & Clarke, 2017). The implications of studies on this species thus complement those in which the consequences of reduced rainfall on terrestrial ecosystems have been explored in the biogeographical hotspot of south‐western Australia (Fitzpatrick, Gove, Nathan, Sanders, & Dunn, 2008).

This study had the following aims. (a) Describe the changes in rainfall during recent decades in the catchments of examples of the various types of estuary, including those selected for determining the biological characteristics of *A. butcheri* in south‐western Australia, and elucidate the degree to which any such changes are reflected in volume of freshwater discharge. (b) Develop a steady‐state oxygen mass‐balance model to demonstrate that declines in freshwater discharge can negatively affect oxygen concentrations in the different types of estuaries studied. (c) Determine the growth and body condition of *A. butcheri* in six estuaries in 2013–15 to illustrate the extent to which these key biological variables vary among estuaries and whether there are indications that such variations are influenced by differences in temperature and salinity. (d) Compare biological data for *A. butcheri* in four of the estuaries in 2013–15 with those for the same estuaries in 1993–96, for which there are historical biological data (Sarre & Potter, 1999, 2000). These comparisons were used to explore the hypothesis that reductions in freshwater discharge between the two periods were accompanied in *A. butcheri* by declines in growth, body condition, and length at maturity and increases in the age at maturity.

## 2 MATERIALS AND METHODS

### 2.1 Rainfall and freshwater discharge

**Figure 1:**
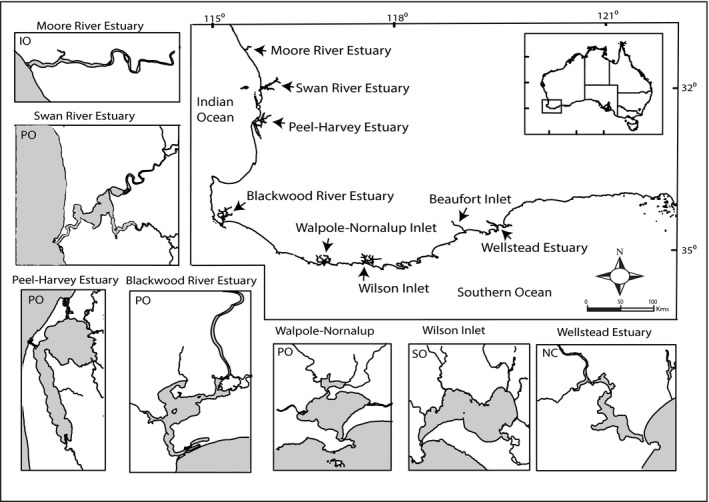
Map showing locations in south‐western Australia of the six estuaries in which *Acanthopagrus butcheri* was sampled by seine and gill nets in 2013–15. Freshwater discharge are provided in Figure 2 for these estuaries and the Blackwood River Estuary. Location of Beaufort Inlet, which acted as a surrogate for Wellstead Estuary for both discharge and used in the hydrological model, is also shown. Estuaries and ocean are shaded. IO: intermittently‐open; NC: normally‐closed; PO: permanently‐open; SO: seasonally‐open

The locations of the estuaries, for which environmental and/or biological data were recorded between 1990 and 2015, are shown in Figure 1. Annual rainfalls were obtained from the Australian Bureau of Meteorology (2018b) for the following stations (a) Gingin in the catchment of the Moore River Estuary, (b) Perth Airport in the catchment of the Swan River Estuary, (c) Dwellingup in the catchment of the Peel‐Harvey Estuary, (d) Cape Leeuwin, adjacent to the Blackwood River Estuary, (e) Vermeulen in the catchment of the Walpole‐Nornalup Inlet, (f) Albany, near Wilson Inlet, and (g) Jeramungup in the catchment of the Wellstead Estuary, near Beaufort Inlet. The weather station at each location was chosen as it yielded annual rainfall based on an entirely or largely continuous series of daily values. Annual freshwater discharges in the above estuaries, among which Beaufort Inlet acted as a surrogate for the nearby Wellstead Estuary for which there were no such data, were obtained from the Western Australian Department of Water (2017).

A log‐log equation (using natural logarithms) describing the relationship between annual values for discharge in each estuary and rainfall, at the selected weather station in its catchment in the years between 1990 and 2015, was fitted and tested for significance. A linear equation was fitted to describe, for each weather station, the relationship between rainfall and the years between 1990 and 2015, while a log‐linear equation was used to describe, for each estuary, the relationship between freshwater discharge and those years. One‐tailed t tests were employed to determine whether Pearson’s correlation coefficients between annual rainfall and year at the selected stations, and between the natural logarithm of annual freshwater discharge and year in each estuary, were negative and statistically significant (*p *≤ 0.05). Back‐transformed values for predictions of freshwater discharge calculated using the log‐log and log‐linear models were corrected for the bias associated with log transformation (Beauchamp & Olson, 1973).

### 2.2 Oxygen mass‐balance box model

A model was developed to describe the relationship between oxygen concentration throughout the water column and freshwater discharge into an estuary. This model provides a mechanistic description of the expected relationship between declining freshwater discharge and dissolved oxygen concentrations, rather than producing precise estimates of inter‐annual variations in oxygen concentration over time. The model incorporates key processes that influence oxygen concentration, including freshwater and tidal flushing, phytoplankton production and respiration, rates of sediment oxygen demand and air‐water exchange and, in estuaries that become closed for a period, the duration of closure of their mouths. A full description of the model is provided in the Supporting Information Appendix S1.

The mass‐balance model was first applied using values of input parameters collated for the Swan River Estuary (see Supporting Information Appendix S1) and model outputs validated by comparison with dissolved oxygen concentrations recorded in that estuary in each week for the 8 years from 2008 to 2015. Because the climate, and thus hydrology of south‐western Australian estuaries, undergoes highly seasonal changes, the model for the Swan River Estuary was run separately using parameters reflecting summer (e.g., low inflows and high temperatures) and winter conditions (e.g., high inflows and low temperatures). The model was subsequently employed using values of input parameters for summer and winter conditions for the Moore River Estuary and the Walpole‐Nornalup and Beaufort inlets, the last acting as a surrogate for the nearby Wellstead Estuary for which there are no freshwater discharge data but which experiences similar rainfall (Australian Bureau of Meteorology, 2018a). As there were no long‐term monitoring data of oxygen concentration to validate the results for these three estuaries, the model is applied to each estuary using the available setup information without further calibration. Due to uncertainty in forcing conditions and model parameters, a Monte Carlo sensitivity assessment was performed for each application to provide an “envelope” of likely oxygen concentrations for each estuary over a realistic range of conditions (Supporting Information Appendix S1).

### 2.3 Sampling regime for *Acanthopagrus butcheri*

*Acanthopagrus butcheri* was collected by seine and gill netting throughout the upper regions of the Moore River Estuary, Swan River Estuary, Peel‐Harvey Estuary, Walpole‐Nornalup Inlet, Wilson Inlet and Wellstead Estuary (Figure 1). Samples were obtained from several widely‐distributed sites in nearshore, shallow (<2 m) waters using 21.5 and 41.5 m long seines and in deeper (2–6 m) waters, further from the shore, employing a 160 m long composite gill net. Sampling was undertaken in autumn and spring of both 2013 and 2014 and in summer of 2015. Rod and line fishing were used to catch additional *A*. *butcheri* from each estuary on each sampling occasion. Further samples were obtained from the Swan River and Peel‐Harvey estuaries using the same seine and gill nets between autumn 2013 and summer 2015.

The 21.5 m seine, which consisted of a 1.5 m wide bunt of 3 mm mesh and two 10 m long wings (each comprising 4 m of 3 mm mesh and 6 m of 9 mm mesh), swept an area of 116 m^2^, while the 41.5 m seine, which contained a 1.5 m wide bunt made of 9 mm mesh and two 20 m long wings comprising 25 mm mesh, swept an area of 274 m^2^. The 21.5 m seine was laid parallel to the shore and then hauled onto the bank, whereas the 41.5 m seine was deployed in a semi‐circle from the bank, using a small boat, and then hauled on to the bank. The composite gill net, which comprised eight 20 m long panels, each with a different stretched mesh size, that is, 38, 51, 63, 76, 89, 102, 115 and 127 mm, was set at sunset, parallel to the shore and in water depths >2 m, and retrieved 3 hr later. All fish were euthanized in an ice slurry immediately after capture.

Samples of *A. butcheri* had previously been collected, between 1993 and 1996, from the Moore River Estuary, Swan River Estuary, Wellstead Estuary and Walpole‐Nornalup Inlet using seine and gill nets with essentially the same dimensions and mesh sizes as those recorded above (Sarre & Potter, 2000).

### 2.4 Growth and mass‐length relationships

Each *A. butcheri* was measured to the nearest 1 mm (total length), weighed to the nearest 1 g and sexed. The smallest fish, which could not be sexed, were designated randomly (and in equal numbers) as either females or males. The sagittal otoliths of each *A. butcheri* were removed and examined whole using a dissecting microscope under reflected light. The opaque zones were counted in whole otoliths when their number was ≤6 and in sectioned otoliths when their number was greater (see Sarre & Potter, 2000), with the counts then used to age the fish. Full details of the aging procedures are given in Cottingham et al. (2014).

The von Bertalanffy growth model was fitted separately to the lengths at age of females and males of *A. butcheri* in each of the six estuaries sampled in 2013–15, and in the four of those estuaries for which data had been collected in 1993–96. The von Bertalanffy growth equation is ${\hat{L}}_{t}\;=\;L_{\infty }\left[1-\exp\left(-k\left(t-t_{0}\right)\right)\right]$, where ${\hat{L}}_{t}$ is the expected total length at age *t* (years), $L_{\infty }$ is the asymptotic length (mm), *k* is the growth coefficient (per year) and *t*_0_ is the hypothetical age (years) at which fish would have zero length. Likelihood‐ratio tests (see Cerrato, 1990) were used to determine whether the growth curves of both the females and males of *A. butcheri* in the Moore River Estuary, Swan River Estuary, Walpole‐Nornalup Inlet and Wellstead Estuary in 2013–15 differed from those of the corresponding sex in 1993–96.

The age at which, on the basis of the fitted von Bertalanffy growth equation, individuals attained a predicted length equal to the minimum legal length of 250 mm for retention (MLL) in Western Australia, subsequently referred to as “age at 250 mm,” was calculated. These calculations were undertaken for females and males in each of the six estuaries sampled in 2013–15 and in the four of those estuaries sampled in 1993–96.

A preliminary exploration of the mass‐length relationship of each sex of *A. butcheri* in the six estuaries studied in 2013–15, and in the four of those for which there were also data for 1993–96, was undertaken. Following the approach of Froese (2006), a linear equation was fitted to the log‐transformed mass and length of ten randomly‐selected fish from each of three length categories, that is, <160, 180–220, >240 mm, in both autumn and spring. These two seasons were selected because data were available for *A. butcheri* in each estuary in those seasons in both of the above periods. The equation, which was fitted using least squares regression, was ln*M* = a + *b*ln*L*, where ln is the natural logarithm, *M* and *L* are the estimated body mass (g) and total length (mm) of fish, respectively, and *a* and *b* are constants.

As likelihood‐ratio tests demonstrated that the slope (*b*) of the log‐transformed mass‐length relationship for each sex of *A. butcheri* in each estuary in 1993–96 often differed significantly from that of the same sex in that estuary in 2013–15, it was inappropriate to calculate a single overall mean condition factor across the full length range for comparing the body condition of *A. butcheri* in the two periods (Jennings, Kaiser, & Reynolds, 2001). Thus, comparisons of condition between periods used the mass at a given reference length of 250 mm TL, which was chosen as it represented a substantial length achieved after several years of growth in each estuary and is the MLL for *A. butcheri* in south‐western Australia and thus relevant to fisheries management. A reparameterized form of the mass‐length relationship, that is, ln *M* = ln*M*_250 mm_ + *b*ln[*L*/*L*_250 mm_], was therefore fitted to the data for each sex in each estuary and period using the data employed in the preliminary exploration of mass‐length relationships. The estimates of *M*_250 mm_ for each sex in each estuary in the two periods were then compared using a likelihood‐ratio test. For this, the log‐likelihood of a fitted model, which assumed that *M*_250 mm_ was the same for an estuary in both periods, was compared with that of a model that assumed different values of *M*_250 mm_ in those two periods.

The relationships between natural logarithms of the expected mass at 250 mm and age at that length of both the females and males of *A. butcheri* were derived using data for 1993–96 and 2013–15 in the four estuaries for which there were such historical data and for the other two estuaries for which there were data for just the later period.

### 2.5 Age and length at maturity

Each *Acanthopagrus butcheri* caught in the four estuaries in 1993–96 and six estuaries in 2013–15 was assigned, on the basis of the macroscopic appearance of its gonads, a maturity stage (see Sarre & Potter, 1999). As insufficient *A. butcheri* were caught during the spawning seasons in 2013–15 to calculate the ages and lengths at maturity for *A. butcheri* in the Swan River and Wellstead estuaries, these data were augmented by those collected previously for the Swan River Estuary in 2007–11 (Cottingham et al., 2014) and for the Wellstead Estuary in 2005–07 (Chuwen, 2009), respectively. Logistic regression analysis was used to determine the probability *p* that a female or male of a given age possessed maturing or mature gonads during the spawning season in each estuary and period. The logistic equation $p=1/\{1+e^{-s(A-A_{50})}\}$ was fitted separately to the data for females and males for each estuary and period (1993–96 and 2013–15) using R (R Core Team, 2017). In this equation, *A* is the age of the fish in years and *A*_50_ is the age by which 50% of fish have attained maturity. The slope *s* was constrained to lie between 0 and *s*_max_ = 0.2 years when fitting this model, through reparameterization using a value *x*, where *s* = *s*_max_/(1 + *e* ^−*x*^). Approximate confidence limits for each *A*_50_ were calculated from its profile likelihood (Hilborn & Mangel, 1997). The resultant curve for each sex in each estuary in 2013–15 was then compared with the corresponding curve for that sex and estuary in 1993–96 by fitting the equation $p=1/\{1+e^{-\left(s_{1}\left(1-T\right)+s_{2}T\right)\left(A-\left(A_{50}+DT\right)\right)}\}$, where period *T* = 0 for data collected in 1993–96 and *T =* 1 for 2013–15, *s*_1_ is the slope in 1993–96, *s*_2_ is the slope in 2013–15, *A*_50_ represents the value for that parameter in 1993–96 and *D* is the difference between the age at 50% maturity between the second and first periods, that is, the increase in that age for the later period. Confidence limits for *D* were calculated at the 95%, 99% and 99.9% levels using its profile likelihood and examined to determine whether the resulting confidence regions lay above, below or overlapped 0, and thereby assess the probability that *A*_50_ for the later period significantly exceeded that for the earlier period. The same approach, but with length substituted for age, with *s*_max_ constrained to 10 mm, was used to relate the probability that a female or male possessed mature gonads to the total length (mm) of the fish in each estuary and period and to assess whether the *L*_50_ decreased significantly between the two periods.

## 3 RESULTS

### 3.1 Rainfall and freshwater discharge

**Figure 2:**
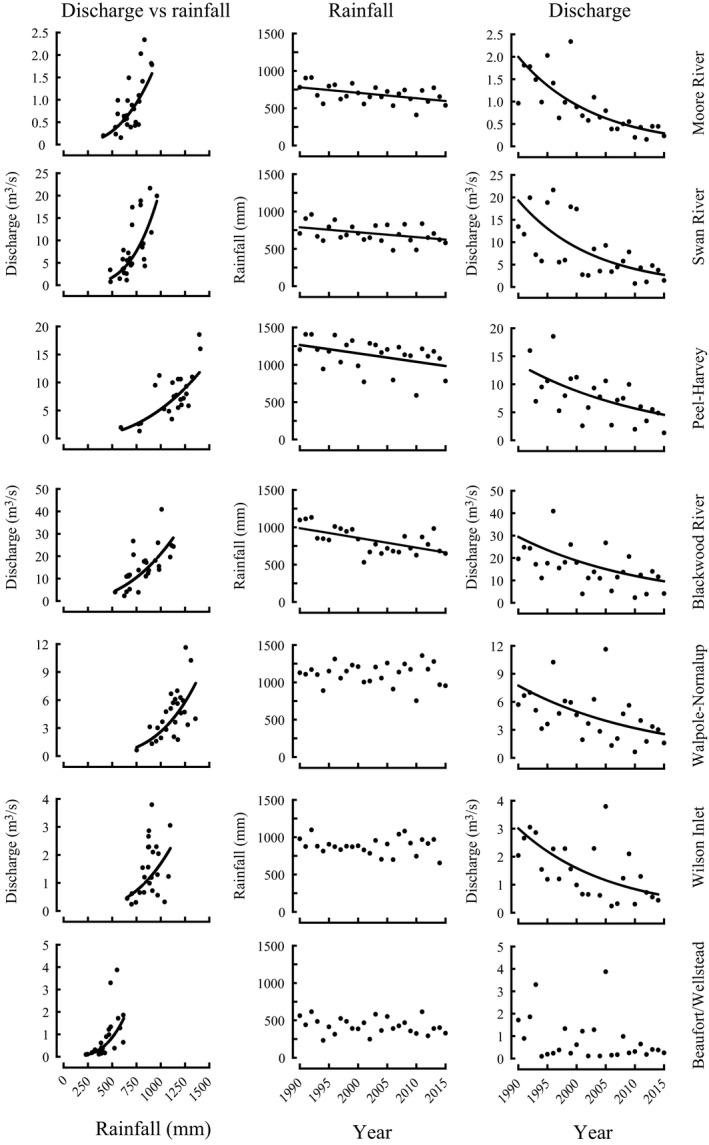
Relationship for annual discharge versus rainfall, and annual rainfall and annual discharge versus year between 1990 and 2015 for the seven estuaries shown in Figure 1. Data were extracted from the websites of the Australian Bureau of Meteorology (2018b) and Western Australian Department of Water (2017)

Annual freshwater discharge in each of the seven estuaries studied throughout south‐western Australia in the years between 1990 and 2015 was significantly related to annual rainfall at the weather station in the catchment of that estuary (Figure 2, Table 1). During that period, annual rainfall declined significantly at each station in the catchments of the four estuaries on the west coast (Figure 2, Table 1). These declines in rainfall parallel those for other nearby weather stations for which there were substantial but incomplete daily data, such as for Midland in the Swan River Estuary catchment. From the regression equations relating rainfall to year, the declines in rainfall between 1990 and 2015 in the catchments of the above four estuaries ranged from 21% at Perth Airport to 33% at Cape Leeuwin. While rainfall in that period declined in the catchments of the Nornalup‐Walpole, Wilson and Beaufort inlets, these declines were only 3, 4, and 18%, respectively, and not significant (Figure 2, Table 1).

**Table 1:** Linear relationships between natural logarithms of freshwater discharge (*D* as m^3^/s) and rainfall (*R*), rainfall and year (*Y*), and between natural logarithm of freshwater discharge and year for the seven rainfall stations/estuaries in south‐western Australia between 1990 and 2015. Discharges into Beaufort Inlet were used as surrogates for those into Wellstead Estuary. Rainfall and discharges were extracted from the websites of the Australian Bureau of Meteorology (2018b) and Western Australian Department of Water (2017), respectively. *r*, Pearson’s correlation coefficient

	Relationship	*r*	*p*
Estuary/rainfall station			
Moore River/Gingin	ln(*D*) = 2.773ln*R *− 18.44	0.71	<0.001
Swan River/Perth Airport	ln(*D*) = 3.650ln*R *− 22.13	0.72	<0.001
Peel‐Harvey/Dwellingup	ln(*D*) = 2.420ln*R *− 15.06	0.79	<0.001
Blackwood/Cape Leeuwin	ln(*D*) = 2.410ln*R *− 13.60	0.70	<0.001
Walpole/Vermeulen	ln(*D*) = 3.560ln*R *− 23.63	0.73	<0.001
Wilson/Albany	ln(*D*) = 2.944ln*R *− 19.81	0.48	<0.05
Beaufort/Jerramungup	ln(*D*) = 3.218ln*R *− 20.15	0.78	<0.001
Rainfall station			
Gingin	*R *= −7.31*Y* + 15318	−0.47	<0.01
Perth Airport	*R *= −6.57*Y* + 3862	−0.41	<0.05
Dwellingup	*R *= −11.25*Y* + 23653	−0.41	<0.05
Cape Leeuwin	*R *= −13.03*Y* + 26920	−0.60	<0.001
Vermeulen	*R *= −1.14*Y* + 3399	−0.06	<0.05
Albany	*R *= −2.21*Y* + 5315	−0.15	>0.05
Jerramungup	*R *= −3.30*Y* + 7042	−0.24	>0.05
Estuary			
Moore River Estuary	ln(*D*) = −0.0770*Y* + 154	−0.83	<0.001
Swan River Estuary	ln(*D*) = −0.0784*Y *+ 159	−0.67	<0.001
Peel‐Harvey Estuary	ln(*D)* = −0.0437*Y* + 89	−0.49	<0.05
Blackwood River Estuary	ln(*D)* = −0.0448*Y* + 92	−0.54	<0.01
Walpole‐Nornalup Inlet	ln(*D*) = −0.0445*Y* + 90	−0.51	<0.01
Wilson Inlet	ln(*D*) = −0.0637*Y* + 128	−0.53	<0.01
Beaufort Inlet	ln(D) = −0.0418*Y* + 83	−0.30	>0.05

Between 1990 and 2015, annual freshwater discharge into the four estuaries on the west coast and two of the three estuaries on the south coast declined exponentially and significantly with year (Table 1, Figure 2). The decline in discharge in the four west coast estuaries ranged from 62% for the Peel‐Harvey Estuary to 89% for the Swan River Estuary. Although discharge did not decline significantly between 1990 and 2015 in Beaufort Inlet, the discharges in three of the first four of those years were among the four highest recorded during the 26 years.

### 3.2 Modelling of oxygen concentrations

**Figure 3:**
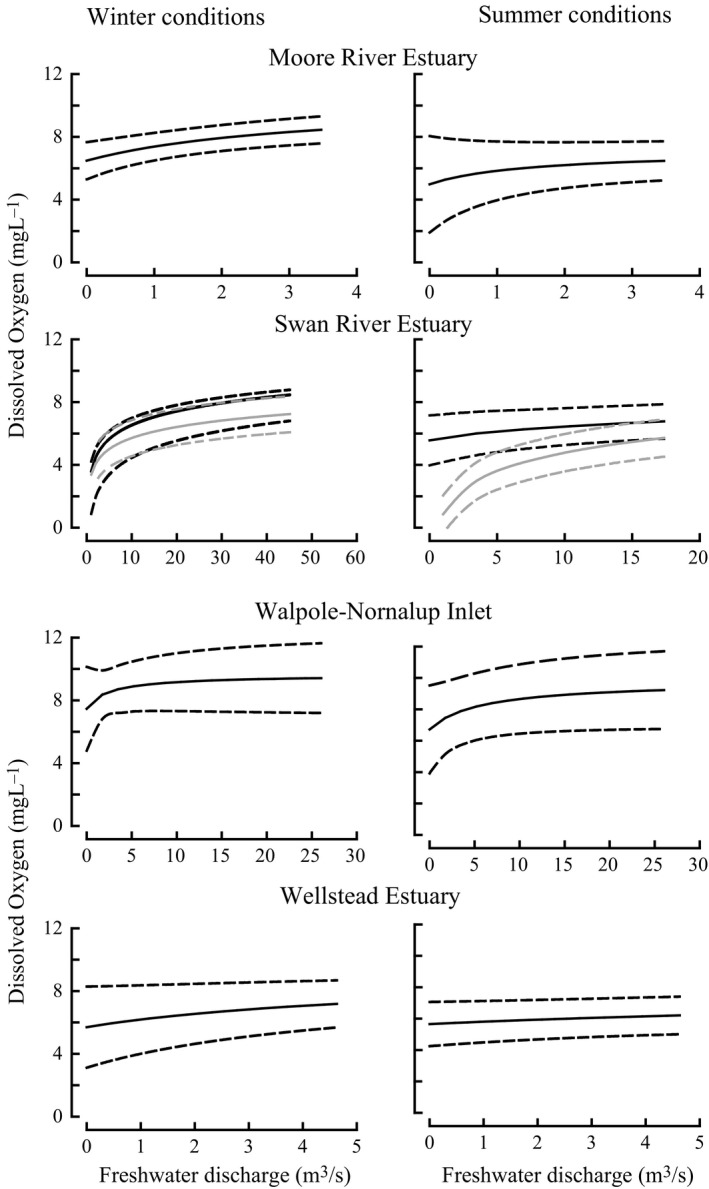
Relationship between freshwater discharge and mean oxygen concentrations ±1 SD throughout the water column in four estuaries under winter and summer conditions predicted from the oxygen mass‐balance model. Gray lines for Swan River Estuary represent mean oxygen concentrations ±1 SD of the bottom waters. See Supporting Information Appendix S1 for model description, parameters, and relationship between dissolved oxygen concentrations of water column and bottom water

The oxygen model predicts that, in both summer and winter, mean oxygen concentrations throughout the water column of the Moore River, Swan River and Wellstead estuaries and Nornalup‐Walpole Inlet decline as freshwater discharge decreases (Figure 3) and that this occurs even though the area, water depth and biogeochemical parameters in these estuaries differ. The shallower estuaries (Moore River and Wellstead) are predicted to be more resilient to reductions in inflow rates, with mean oxygen concentrations in the deeper estuaries (Swan River and Walpole‐Nornalup) declining more markedly when inflow rates become particularly low. Furthermore, reductions in flow result in a greater rate of decline in dissolved oxygen concentrations in bottom waters than in the whole water column. This is demonstrated by the trend exhibited in the Swan River Estuary, based on weekly field measurements that were not available for the other estuaries (Supporting Information Figure S2).

### 3.3 Comparisons of growth and body condition among estuaries in 2013–15

**Figure 4:**
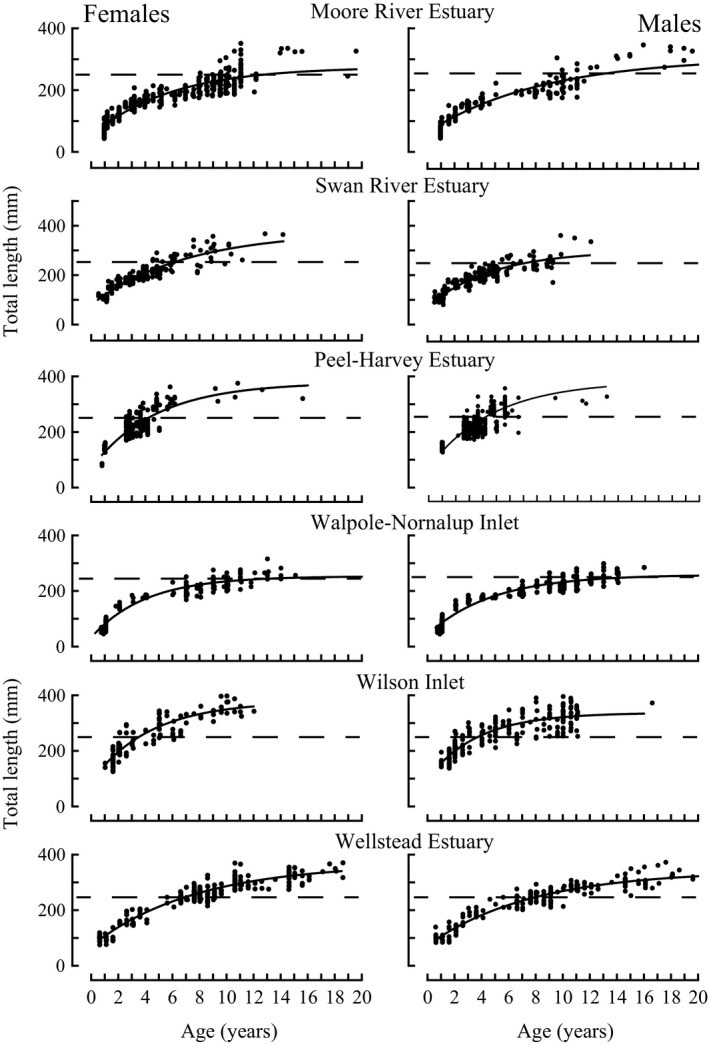
von Bertalanffy growth curves fitted to the lengths at age of female and male *Acanthopagrus butcheri* in 2013–15 in the six estuaries studied in south‐western Australia. Dashed line represents the minimum legal length of 250 mm for retention by fishers

von Bertalanffy growth curves for both the females and males of *A. butcheri* in six estuaries in 2013–15 provide a good fit to the vast majority of the lengths at age of those sexes in those systems (Table 2; Figure 4). The coefficients of determination were always >0.80 and often 0.90 for the curves for both sexes in each estuary except for those of the Peel‐Harvey Estuary (Table 2). The lower coefficients for the curves for females and males in the latter estuary than in the other estuaries are due to the ages of the majority of fish occurring over a more restricted range (Figure 4).

**Table 2:** von Bertalanffy growth curve parameters and 95% confidence limits for females and males of *Acanthopagrus butcheri* caught in the six south‐western Australian estuaries in 2013–15 and in 1993–96 when data were available. *L*_∞_, asymptotic total length (mm); *k*, growth coefficient (per year); *t*_0_, hypothetical age (years) at which fish would have zero length; *r* ^2^, coefficient of determination; *n*, the number of fish. *A*_250 mm_ refers to age (years) at which the predicted length of 250 mm is attained and *M*_250 mm_ is expected mass (g) at 250 mm

Estuary	Latitude	Period	Sex	Statistic	von Bertalanffy growth model					*A*_250 mm_	*M*_250 mm_
					*L*_∞_	*k*	*t*_0_	*n*	*r* ^2^		
Moore	31.37°S	1993–96	Female	Mean	494	0.09	−0.81	304	0.92	7.0	256
				Lower	442	0.08	−1.03				
				Upper	545	0.11	−0.59				
			Male	Mean	473	0.09	−1.01	342	0.91	7.3	268
				Lower	422	0.07	−1.27				
				Upper	523	0.11	−0.76				
		2013–15	Female	Mean	286	0.16	−1.33	398	0.85	11.6	243
				Lower	276	0.09	−1.74				
				Upper	335	0.15	−0.91				
			Male	Mean	313	0.11	−2.19	324	0.85	12.4	253
				Lower	284	0.08	−2.76				
				Upper	344	0.13	−1.61				
Swan	31.94°S	1993–96	Female	Mean	441	0.29	−0.12	680	0.94	2.8	278
				Lower	427	0.27	−0.19				
				Upper	454	0.31	−0.06				
			Male	Mean	424	0.29	−0.19	754	0.99	2.9	283
				Lower	413	0.27	−0.26				
				Upper	436	0.31	−0.13				
		2013–15	Female	Mean	376	0.14	−1.68	193	0.85	6.1	256
				Lower	330	0.10	−2.23				
				Upper	422	0.18	−1.13				
			Male	Mean	288	0.25	−0.75	215	0.83	7.4	263
				Lower	281	0.24	−0.81				
				Upper	296	0.27	−0.70				
Peel‐Harvey	32.62°S	2013–15	Female	Mean	378	0.21	−0.97	185	0.68	4.2	264
				Lower	335	0.15	−1.56				
				Upper	420	0.28	−0.38				
			Male	Mean	382	0.16	−1.99	417	0.56	4.7	259
				Lower	320	0.09	−2.73				
				Upper	443	0.21	−1.25				
Walpole‐Nornalup	35.00°S	1993–96	Female	Mean	366	0.17	−0.59	346	0.91	6.2	248
				Lower	352	0.15	−0.82				
				Upper	380	0.19	−0.37				
			Male	Mean	324	0.21	−0.39	265	0.90	6.6	252
				Lower	313	0.19	−0.62				
				Upper	336	0.24	0.15				
		2013–15	Female	Mean	253	0.25	−0.56	168	0.90	17.7	239
				Lower	241	0.20	−0.89				
				Upper	265	0.30	−0.22				
			Male	Mean	259	0.21	−0.85	188	0.90	15.5	244
				Lower	247	0.17	−1.22				
				Upper	270	0.25	−0.48				
Wilson	34.99°S	2013–15	Female	Mean	384	0.22	−1.21	117	0.83	3.6	261
				Lower	346	0.14	−2.06				
				Upper	422	0.31	−0.36				
			Male	Mean	337	0.27	−1.29	220	0.76	3.7	262
				Lower	320	0.19	−2.03				
				Upper	353	0.35	−0.56				
Wellstead	34.39°S	1993–96	Female	Mean	378	0.26	−0.25	357	0.94	3.9	237
				Lower	366	0.24	−0.33				
				Upper	390	0.27	−0.18				
			Male	Mean	344	0.27	−0.25	358	0.95	4.6	240
				Lower	336	0.26	−0.32				
				Upper	353	0.29	−0.18				
		2013–15	Female	Mean	367	0.13	−1.65	301	0.91	7.1	249
				Lower	354	0.11	−2.00				
				Upper	384	0.14	−1.31				
			Male	Mean	342	0.13	−1.84	212	0.91	8.3	256
				Lower	326	0.11	−2.27				
				Upper	358	0.15	−1.40				

**Figure 5:**
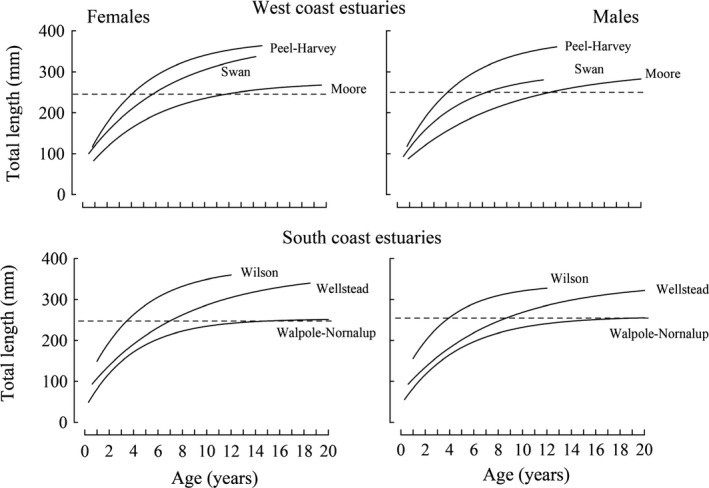
Comparisons of von Bertalanffy growth curves for female and male *Acanthopagrus butcheri* in estuaries on the west and south coasts of south‐western Australia in 2013–15. Dashed line represents the minimum legal length of 250 mm for retention by fishers

Pairwise likelihood‐ratio tests demonstrate that, in comparisons between each pair of the six estuaries in 2013–15, the growth curves for the females and males of *A. butcheri* (Figure 5) were both typically significantly different (*p *< 0.01 or 0.001). This is reflected in marked variations in the durations taken by both females and males to reach their respective ages at the MLL of 250 mm (Table 2). Thus, these time spans ranged in females from 3.6 years in Wilson Inlet to as high as 17.7 years in Walpole‐Nornalup Inlet and in males from 3.7 years to as great as 15.5 years in those same two estuaries (Table 2).

The expected body mass of *A. butcheri* at 250 mm in these estuaries in 2013–15 ranged for females from 239 g in the Walpole‐Nornalup Inlet to 264 g in the Peel‐Harvey Estuary and for males from 244 g in Walpole‐Nornalup Inlet to 262 g in Wilson Inlet (Table 2).

### 3.4 Comparisons of growth and body condition in 2013–15 and 1993–96

**Figure 6:**
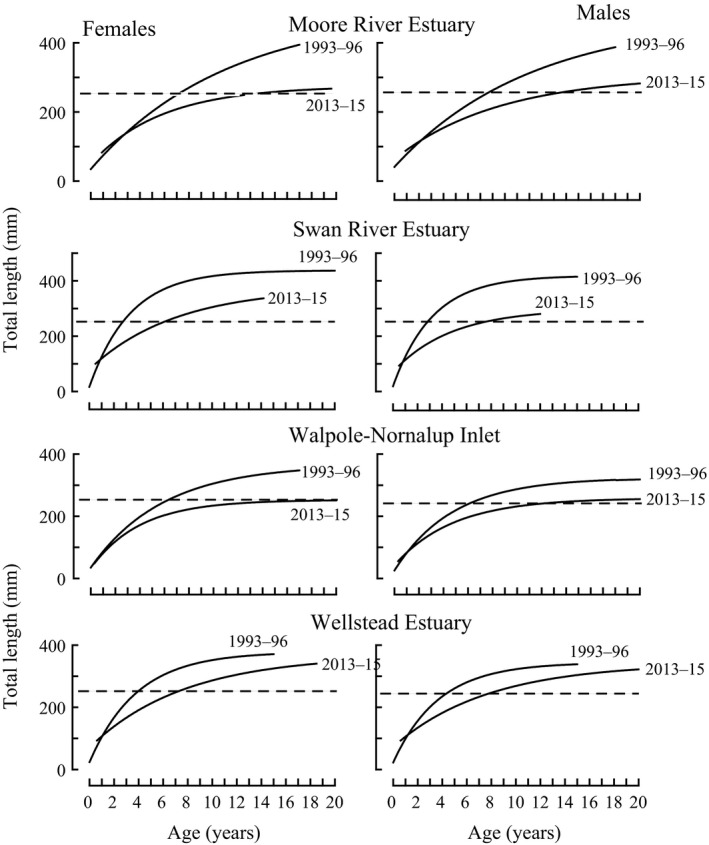
Comparisons of von Bertalanffy growth curves for female and male *Acanthopagrus butcheri* in the Moore River Estuary, Swan River Estuary, Walpole‐Nornalup Inlet and Wellstead Estuary in 1993–96 and 2013–15

The growth curves for both females and males of *A. butcheri* in the Moore River Estuary, Swan River Estuary, Walpole‐Nornalup Inlet and Wellstead Estuary in 2013–15 lay below the corresponding curves for 1993–96 across the vast majority of the age range of the fish (Figure 6). The slight overlap at the very lower end of the age range in these comparisons reflects differences in the estimates for *t*_0,_ rather than intrinsic differences between the growth in the two periods. The growth curves for both females and males in each estuary differed significantly between the two periods (all *p *< 0.001).

The years required for female and male *A. butcheri*, in the Moore River Estuary, Swan River Estuary, Walpole‐Nornalup Inlet and Wellstead Estuary, to reach predicted lengths equal to the MLL of 250 mm increased markedly between 1993–96 and 2013–15, thus delaying recruitment of the corresponding year classes into the fishery (Table 2). This was particularly the case in the Walpole‐Nornalup Inlet in which, on average, females would have taken almost three times as long to reach the MLL in the recent than earlier period (Table 2). Indeed, the slower growth in 2013–15 than in 1993–96 would have led to a proportionately smaller number of individuals surviving for the additional time required to attain the MLL and thus to an overall reduction in recruits into the fishery.

The body mass of both females and males of *A. butcheri* with lengths of 250 mm in the Moore River Estuary, Swan River Estuary, and Walpole‐Nornalup Inlet declined between 1993–96 and 2013–15 (Table 2) and, in each case, the difference was significant (*p *< 0.001 to 0.05). For example, a 250 mm female in the Moore River Estuary weighed, on average, 16 g less in 2013–15 than in 1993–96. In contrast, the masses of both female and male *A. butcheri* of 250 mm in the Wellstead Estuary increased between 1993–96 and 2013–15 (Table 2).

**Figure 7:**
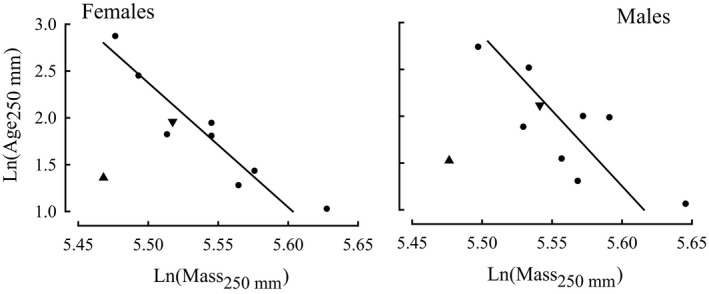
Linear relationships between the natural logarithms (Ln) of the ages at which the predicted lengths of the females and the males of *Acanthopagrus butcheri* are 250 mm and of the body masses of each sex at 250 mm. 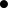 represent points for the four estuaries sampled in 1993–96 (see Figure 6) and the six estuaries sampled in 2013–15 (see Figures 4 and 5), while 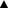 and 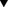 refer to points for Wellstead Estuary in 1993–96 and 2013–15, respectively

Body masses of females and males of *A. butcheri* at 250 mm in the Moore River and Swan River estuaries and Walpole‐Nornalup Inlet were inversely related to the corresponding expected ages at 250 mm in those systems (Figure 7; *p *< 0.001, *r *= −0.947 for females and *p *< 0.01, *r *= −0.787 for males). While the natural logarithms of *M*_250 mm_ and associated *A*_250 mm_ for both females and males in the Wellstead Estuary in 2013–15 lay close to the regression line for the corresponding sex derived using data for the above three estuaries, this did not apply to the corresponding values of those variables for that estuary in 1993–96, and thus constituted conspicuous outliers (Figure 7).

### 3.5 Age and length at maturity

**Figure 8:**
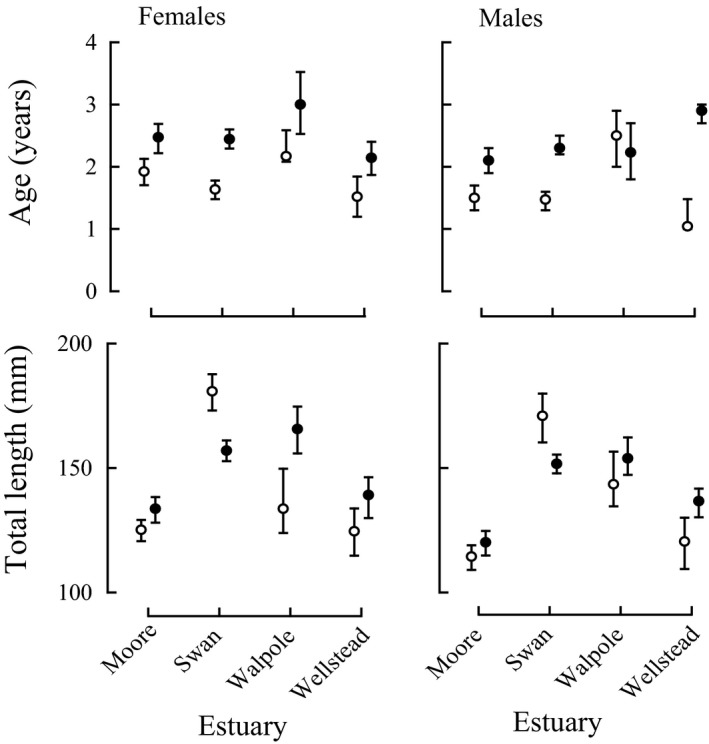
Median values and 95% credible intervals for the ages and total lengths of females and males at maturity in the Moore River, Swan River, and Wellstead estuaries, and the Walpole‐Nornalup Inlet in 1993–95 (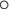) and 2013–15 (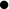)

Among the four estuaries, for which there were historical reproductive data for *A. butcheri*, the age at maturity (*A*_50_) of both the females and the males was significantly greater in 2013–15 than in 1993–96 (*p* < 0.01–0.001) in all cases except for males in the Walpole‐Nornalup Inlet (*p *> 0.05; Figure 8).

Although the lengths at maturity of both the females and males in the Swan River Estuary were greater in 1993–96 than in 2013–15 (*p *< 0.001), the reverse applied to both sexes in each of the other three estuaries, noting that the differences were not significant for males in the Moore River Estuary and Walpole‐Nornalup Inlet.

## 4 DISCUSSION

### 4.1 Rainfall and freshwater discharge: relationships and changes with time

The analyses of data from the Australian Bureau of Meteorology (2018b) and Western Australian Department of Water (2017) confirm that annual discharges into different types of estuaries in south‐western Australia were positively correlated with annual rainfall in their catchments in the years between 1990 and 2015. These estuaries included those for which the biological characteristics of *Acanthopagrus butcheri* were determined, noting that the discharge in Beaufort Inlet was used as a proxy for that in Wellstead Estuary. Discharge has also been found to be positively correlated with rainfall in Stokes Inlet, which is located 170 km to the east of the most eastern system (Wellstead Estuary) considered in the present study (Hoeksema et al., 2018). The inflows into the 11 major dams in the Perth region in the years from 1911 to 1976 and 1977 to 2013 have previously been shown to be positively correlated with rainfall throughout south‐western Australia (Smith & Power, 2014). It is predicted from models that, by 2030, a further decline in rainfall of ~8% will be accompanied by a reduction in discharge of ~25% (Silberstein et al., 2012).

The magnitude of the declines in annual rainfall recorded between 1990 and 2015 at weather stations in the catchments of the four west coast estuaries and three south coast estuaries are consistent with those “modelled” for that region between 1970 and 2018 (Australian Bureau of Meteorology, 2018a). Thus, the percentage declines of 21%–33% in annual rainfall between 1990 and 2015 in the catchments of the four west coast estuaries were typically far greater than the reductions of 3%, 4% and 18% in the catchments of the three south coast estuaries, with the former declines statistically significant.

The percentage declines of 62%–89% in annual freshwater discharge in the four west coast estuaries in 1990–2015 greatly exceed those of annual rainfall in the corresponding catchments of those estuaries (see above). The far greater proportional declines in freshwater discharge are in line with estimates that a decline in rainfall of 15%–20% was accompanied by a reduction of up to 70% in runoff into reservoirs in the region of the Swan River Estuary (Bates et al., 2008; Silberstein et al., 2012). Although a decline in rainwater input via streams and tributaries is the main contributor to declines in freshwater discharge, a reduction in rainfall has also reduced groundwater levels and thus the “reservoir” of supplementary water that contributes to discharge (Smettem, Waring, Callow, Wilson, & Mu, 2013). Furthermore, declines in freshwater discharge have almost certainly been exacerbated by factors, such as the diversion of water for agriculture, industry and human use (McFarlane et al., 2012; Mehran et al., 2017; Stewardson et al., 2017). In south‐western Australia, these effects would have been most pronounced in the regions of the Peel‐Harvey and Swan River estuaries, which are well‐populated and extensively developed for agriculture (McFarlane et al., 2012). The progressive downward trend in groundwater levels of the Gnangara Mound in the catchment of the Swan River Estuary between 1997 and 2017 reflects a combination of the effects of declining rainfall and increasing water abstraction (http://www.water.wa.gov.au/water-topics/groundwater/understanding-groundwater/gnangara-groundwater-system).

The finding that, on the south coast, the declines in annual rainfall on the Frankland River, which discharges into the Walpole‐Nornalup Inlet, and at Albany near Wilson Inlet were not statistically significant is broadly consistent with Albany lying in a region where, between 1970 and 2017, rainfall declined only slightly (Australian Bureau of Meteorology, 2018a). The highly significant relationships between discharges in the rivers entering both the Walpole‐Nornalup and Wilson inlets and year emphasize that a small and statistically insignificant proportional change in average annual rainfall in the catchment of an estuary can lead to a large and statistically significant proportional change in average freshwater discharge.

### 4.2 Relationship between dissolved oxygen concentrations and freshwater discharge

It was previously established that the spatial extent of hypoxia in the Swan River Estuary increased significantly between 1995 and 2010 as freshwater discharge declined (Cottingham et al., 2014). Although the other estuaries in this study do not possess the same spatio‐temporal pattern of hydrologic dynamics, the oxygen‐balance model still indicates that a reduction in freshwater discharge likewise leads to an increase in retention time and influence of sediment oxygen demand on oxygen concentration in those estuaries and particularly in summer.

Although the magnitudes of predicted reductions in mean oxygen concentrations throughout the water column are modest, the model’s predictions are conservative in that they do not assume periodic stratification (which would occasionally enhance the oxygen drawdown) and, more importantly, do not take into account the localized areas of low oxygen that will develop during warm conditions and low wind. Furthermore, the declining summer base‐flow in key rivers of south‐western Australia is leading to longer periods of very low flow (Smettem et al., 2013). This will increase the extent and duration of hypoxic events in the bottom waters of the upper estuarine reaches, expanding the low oxygen tail on the oxygen probability distribution (i.e., frequency of events below 4 mg/L). In addition, low flow rates in winter lead to a greater accumulation of organic‐rich sediments and thus to an increase in hypoxia in deeper waters, particularly in summer (Cottingham et al., 2014), which is not taken into account by the model. Such accumulations are particularly relevant for demersal species, such as *A. butcheri*, whose larger individuals live in deeper waters when conditions are favorable, but occupy shallow waters when those deeper waters become hypoxic, thus leading to increases in density (Cottingham et al., 2014, 2018). Oxygen concentrations would be less likely to decline markedly in shallow than deeper waters as their oxygen content is overwhelmingly influenced by oxygen air‐water exchange rather than by freshwater discharge. Furthermore, shallow waters are less prone to stratification.

The model, which describes the relationship between oxygen concentrations throughout the water column and flow, predicts that, as a result of a reduction in average inflow rates, dissolved oxygen concentrations in the different types of estuary studied would have declined between 1990 and 2015. This implies that each estuary has been impacted, to some extent, by the reduction in freshwater discharge that occurred during this period. Confirmation of this conclusion requires, however, the acquisition of comprehensive environmental data for those estuaries for which such data are not available.

### 4.3 Comparisons of growth and body condition among estuaries in 2013–15

The biological data derived for *A. butcheri* in 2013–15 in six estuaries, representing a range of estuarine types and environments, demonstrate that, during the same time period, the growth and body condition of this sparid varied greatly among these systems. With respect to growth, it is particularly relevant that the growth rates of *A. butcheri*, cultured separately from broodstock from the Swan River and Moore River estuaries in December 1999, were essentially identical, whereas the growth of the wild stock was far greater in the former estuary (Partridge et al., 2004). This provides strong circumstantial evidence that the growth of *A. butcheri* is related more to the environmental characteristics of an estuary than to the small differences that exist between the genetic compositions of this sparid in different estuaries (Chaplin, Baudains, Gill, McCulloch, & Potter, 1997; Partridge et al., 2004). As *A. butcheri* is a highly opportunistic “feeder” (Sarre et al., 2000), it is thus relevant that this species fed mainly on bivalve molluscs in the Swan River Estuary, compared with large volumes of the macroalgae *Cladophora* sp. in the Moore River Estuary, presumably reflecting differences in the relative abundances of the food sources in those estuaries (Chuwen et al., 2007; Sarre et al., 2000). As the main dietary components of *A. butcheri* are of less nutritional value in the Moore than Swan River Estuary, it was proposed that the differences in dietary composition are considered likely to have contributed to the differences between the growth of *A. butcheri* in these two systems (Sarre & Potter, 2000). Major differences in the dietary composition of *A. butcheri* in a range of estuaries (Chuwen et al., 2007; Sarre et al., 2000) could thus account for the variations exhibited by the growth of this sparid among estuaries (Chuwen, 2009; Cottingham et al., 2014; Sarre & Potter, 2000; present study). These differences demonstrate that *A. butcheri* is highly opportunistic in its “choice” of food. As the density of *A. butcheri* was greater in the Moore than Swan River Estuary, a density‐dependent effect may also have contributed to the differences between the growth of this sparid in these two systems (Partridge et al., 2004; Sarre & Potter, 2000).

It was striking that, in each of the six estuaries studied in 2013–15, the age and mass of *A. butcheri* at the MLL of 250 mm, regarded as a reflection of growth and body condition, respectively, did not follow that of the latitudinal sequence of these estuaries (cf. Figures 1 and 5, Table 2). Thus, for example, growth and body condition were greatest in Wilson Inlet at 34.99°S on the south coast and Peel‐Harvey Estuary at 32.62°S on the west coast and least in the Walpole‐Nornalup Inlet at 35.00°S on the south coast and Moore River Estuary at 31.37°S on the west coast. Although water temperatures in each of these estuaries have not been recorded continuously, such data are available for air temperatures in their regions, whose trends will essentially parallel those of water temperature. The monthly mean maximum air temperatures for the region between the intermittently‐open Moore River Estuary and permanently‐open Peel‐Harvey Estuary at 31.37°S to 32.62°S are greater in each month than in the region between the permanently‐open Walpole‐Nornalup Inlet and normally‐closed Wellstead Estuary at 35.00°S to 34.39°S, with respective ranges of 19–31°C for the west coast and 16–23°C for the south coast (Australian Bureau of Meteorology, 2018b). This implies that, while growth and body condition of *A. butcheri* in south‐western Australian estuaries are strongly correlated, variations in each of these two biotic variables among estuaries are not conspicuously related to temperature or estuary type.

The present and other studies demonstrate that, in south‐western Australia, *A. butcheri* is abundant and can grow in different types of systems and in salinities ranging from close to zero to in excess of that of full strength seawater (e.g., Cottingham et al., 2014; Sarre & Potter, 2000; Young & Potter, 2002; Young, Potter, & de Lestang, 1997). Furthermore, in laboratory trials, this species survived well and grew in salinities ranging from 0 to 48 (Partridge & Jenkins, 2002). The above comparisons emphasize that *A. butcheri* is highly euryhaline. Although *A. butcheri* moves within estuaries and thus encounters a range of salinities, there are no clear indications that the variations in growth among estuaries consistently follow the trends exhibited by the broad salinity regimes in these systems. It is thus concluded that differences in temperature and salinity among estuaries are not primarily responsible for the large variations in the growth of *A. butcheri* among estuaries in south‐western Australia. However, at a far larger spatial scale, the growth of *A. butcheri* was shown to be negatively correlated with temperature in estuaries in the relatively cool climate of Tasmania over a 21 year period and positively correlated with temperature (and also rainfall) in an estuary in South Australia over a 13 year period (Doubleday et al., 2015).

Although there were data that enabled us to propose that differences in the growth of *A. butcheri* among estuaries were not strongly related to either water temperature (with air temperature as a surrogate) or salinity, there are no data to facilitate comparisons of the oxygen regimes across those estuaries. When available, oxygen concentration in those estuaries was based on measurements taken on a single day during sampling and often at seasonal intervals and thus do not provide a reliable basis for comparing oxygen concentrations among those estuaries.

### 4.4 Comparisons of growth, body condition and maturity schedules between periods

This study has confirmed the hypothesis that reductions in freshwater discharge, and thus presumably increases in the extent of hypoxia in the Moore River, Swan River and Wellstead estuaries and Nornalup‐Walpole Inlet between 1993–96 and 2013–15, were accompanied by declines in the growth of *A. butcheri*. The decline in growth was reflected in an increase in the average duration taken by fish to attain a length of 250 mm, with increases by females of ~2 times in the Moore River, Wellstead and Swan River estuaries and as high as ~3 times in the Walpole‐Nornalup Inlet. Since the time taken by males to reach 250 mm in each estuary also increased markedly, and to a similar degree, the same trends were exhibited by both sexes. The decline that consistently occurred in the growth of females and males in each of the above four estuaries between 1993–96 and 2013–15 implies that, throughout the large region of south‐western Australia, this species was responding to factor(s) that were operating in a common direction.

The declines in the growth of both sexes of *A. butcheri* in Moore River Estuary, Swan River Estuary and Walpole‐Nornalup Inlet between 1993–96 and 2013–15 were accompanied by reductions in body condition in all estuaries except Wellstead Estuary. Furthermore, when using data for each sex in both periods in the above four estuaries (except for those for Wellstead Estuary in 1993–96) and those for the Peel‐Harvey Estuary and Wilson Inlet in 2013–15, the mass and age at 250 mm were closely related. This implies that typically the factor(s) regulating the growth and body condition of *A. butcheri* are closely linked and that any tendency for body condition to decline will be accompanied by a reduction in energy reserves that could be used for growth (Lambert & Dutil, 1997; Lloret, Shulman, & Love, 2014; Morgan, 2004; Rӓtz & Lloret, 2003).

The decline that occurred in the growth and typically body condition of *A. butcheri* in estuaries between 1993–96 and 2013–15, as discharge decreased and the extent of hypoxia is thus presumed to have increased, is consistent with the reductions in the values recorded for those biological characteristics in fishes in the northern hemisphere when exposed to hypoxia (e.g., Pichavant et al., 2001; Wu, 2002). In the Neuse River Estuary, the growth and body condition of the Atlantic Croaker *Micropogonias undulates* was least in years when hypoxia was greatest (Eby et al., 2005), a trend consistent with the results of subsequent laboratory experiments (Mohan, Rahman, Thomas, & Walther, 2014). Furthermore, hypoxic events, such as those that occur in the Neuse River Estuary and Swan River Estuary, have been shown to have deleterious effects on the prey resources of demersal fish (Powers et al., 2005; Tweedley, Hallett, Warwick, Clarke, & Potter, 2016). Thus, for example, following hypoxic events, the abundance of the clam *Macoma balthica* declined by 90% in the Neuse River Estuary and molluscs and crustaceans virtually disappeared in the Swan River Estuary (Powers et al., 2005; Tweedley, Hallett et al., 2016). In addition, the decline in environmental quality in different south‐western Australian estuaries has been accompanied by the same directional changes in taxonomic distinctness of benthic macroinvertebrates (Tweedley et al., 2014), key prey of *A. butcheri* in this region (Chuwen et al., 2007; Sarre et al., 2000).

The question now arises as to why the growth of both sexes of *A. butcheri* in each of four estuaries declined between periods but did not likewise occur with body condition in one of those estuaries, that is, Wellstead Estuary. As the point for the mass and age at 250 mm for both the females and males in Wellstead Estuary in 1993–96 lies well below the regression line relating mass and age of each sex at 250 mm across estuaries and periods, the body condition of *A. butcheri* in that estuary was atypically low in 1993–96. It therefore appears pertinent that, due to its isolation from the sea and limited freshwater input, Wellstead Estuary became markedly hypersaline for an interval in that period and that *A. butcheri* becomes osmotically stressed when salinities reach 60 (Hoeksema, Chuwen, & Potter, 2006; Partridge & Jenkins, 2002; Young & Potter, 2002). While relatively transient, but extreme, environmental changes may, therefore, have deleteriously affected body condition on a temporary basis, they would have had far less influence on overall growth, as growth curves are calculated using lengths at age, which, particularly in older fish, represent a cumulative effect over a protracted time scale. It is thus particularly pertinent that the *A. butcheri* caught in the Wellstead Estuary in 1993–96 were overwhelmingly dominated by the 1991–1993 year classes and that these years corresponded to those when freshwater discharge was typically greatest. This would account for the growth of both sexes in the Wellstead Estuary declining between 1993–96 and 2013–15 as the environment deteriorated and thus parallel the trends in other estuaries as the extent of hypoxia likewise increased.

As the marked variations in the growth and body condition of *A. butcheri* across a wide range of estuaries in the same time period, 2013–15, were not conspicuously related to temperature and salinity, it appears unlikely that the significant changes in growth and body condition, which occurred between 1993–96 and 2013–15 in the four estuaries for which there were historical biological data, were closely related to the extent of the changes in these variables between the two periods. For example, in the Swan River Estuary, the annual mean for monthly maximum air temperatures, derived from continuous daily measurements at Perth Airport in the vicinity of the upper region of that estuary, in which *A. butcheri* spends the majority of its life, increased, on average of only ~1.8°C between 1990 and 2015 (Cottingham et al., 2016). Furthermore, the corresponding increase in water temperature between these periods, for which there are no directly comparable data, would be expected to be even less. On the basis of the regression equation relating salinity to year and derived from weekly measurements between 1995 and 2015, the salinity at a site in the Swan River Estuary, increased, on average, by 5.8 at the surface and 8.3 at the bottom of the water column (Water Information, 2018), with surface and bottom salinities at all times well within the range in which *A. butcheri* is known to grow well (Partridge & Jenkins, 2002).

The increase in age at maturity that typically occurred as growth declined implies that *A. butcheri* now requires a greater period to attain the threshold length necessary for maturation, which is consistent with changes predicted by life‐history theory (e.g., Plaistow, Lapsley, Beckerman, & Benton, 2004; Stearns & Koella, 1986) and exhibited in wild populations, such as of the Chum Salmon *Oncorhynchus keta* in northern Japan (Morita & Fukuwaka, 2007). In contrast to life‐history theory, the length at maturity of *A. butcheri* declined in only one of the four estuaries. As growth in the other three estuaries was not as great as in that estuary, it is proposed that the individuals in those estuaries require longer to attain a threshold size for becoming mature and thus, through further growth prior to maturity at the commencement of the following or later spawning season(s), lengths at maturity have become greater.

It is proposed that the changes in the biological characteristics of *A. butcheri* in south‐western Australian estuaries during recent years are predominantly related to the effects of hypoxia directly on the metabolism of this species and indirectly through leading to changes to particularly the composition of potential food sources and increases in density through habitat compression.

This study emphasizes that the management of the different types of estuaries in south‐western Australia needs to take into account that declining rainfall has been accompanied by large reductions in freshwater discharge into those systems, a trend which, in populated and agricultural areas, would have been exacerbated by anthropogenic influences such as abstraction. The results and modelling imply that consequent reductions in flushing have led to increased accumulations of nutrients and organic material and thus in the extent of hypoxia, thereby leading to detrimental changes to the biological characteristics of *A. butcheri*. Every attempt should thus be made to maintain current discharge levels and, where possible, reduce the detrimental effects of anthropogenic activities on flow within the estuary. From a fisheries management perspective, the reduced growth and changes in maturity schedules of *A. butcheri* will probably have reduced recruitment to the fishery and/or spawning biomasses of the populations in the different south‐western Australian estuaries and thus the amenity to fishers of this important recreational fish species in those estuaries. These detrimental biological effects will presumably worsen as a result of the effects of continuing climatic changes (Barron et al., 2012). It thus follows that ongoing assessment of the biological characteristics of highly adaptable species, such as *A. butcheri*, would provide an excellent additional method for monitoring and assessing the “health” of estuaries.

## CONFLICT OF INTEREST

None declared.

## AUTHOR CONTRIBUTIONS

Alan Cottingham (AC), Ian C. Potter (ICP) and Matthew Hipsey (MH) developed initial concepts for the study. Eloise Ashworth and Joel Williams collected samples, aged fish, and staged their gonads. MH and Peisheng Huang developed the mass‐balance model and, together with AC, applied it to data for the different estuaries. AC and Norm Hall (NH) performed statistical analyses. AC, PH, MH, NH, and ICP wrote and edited the manuscript. All authors reviewed drafts of the manuscript and read and approved the final version.
