# Supplementary material for: Growth, condition, and maturity schedules of an estuarine fish species change in estuaries following increased hypoxia due to climate change
Source: Ecol Evol. 2018 Jun 25;8(14):7111–30. doi: 10.1002/ece3.4236 (PMC6065278; doi:10.1002/ece3.4236)
Supplement: Supplementary file 3 [file ECE3-8-7111-s003.docx]

**Supporting Information 1: Estuary Oxygen Mass Balance Model**

## 1.1 Model description

A steady-state, mass-balance oxygen model is applied to estimate the relationship between dissolved oxygen concentration in each estuary ($O_{2,E}$) and river inflow rate (*V*_I_). The oxygen model is based on the box model described in Adiyanti & Hipsey (2015), with simplifications to exclude the processes of wrack biochemical oxygen demand and groundwater input. The rate of change of $O_{2,E}$ is calculated as

(1) $\frac{dO_{2,E}}{dt}=F_{o}+F_{I}+P-R-SOD\pm Atm$ , where the variables on the right-hand side of this equation represent the rates of change in oxygen due to the following processes: *F*_o_ = ocean flushing, *F_I_* = freshwater discharge, *P =* phytoplankton production, *R* = phytoplankton respiration, *SOD* = sediment oxygen demand and *Atm* = atmospheric exchange. These latter rates were estimated as described below, where parameter meanings and settings are given in Tables S1, S2. A steady state estimate of $O_{2,E}$ is calculated by solving the equation $dO_{2,E}/dt\equiv0$.

**Ocean Flushing (*F_O_*):**

The dissolved oxygen concentration in the estuary due to mixing between ocean and estuarine water is estimated as

(2) $O_{2,mix}=(k_{O}{\cdot O}_{2,O})+(1-k_{O})\cdot O_{2,E}$ , where

(3) $k_{O}=V_{T}/V_{E}$ , where

(4) $V_{T}=0.5\cdot AMP\cdot A\cdot(1-B)$ .

The rate of change of dissolved oxygen in the estuary due to ocean flushing is therefore

(5) $F_{O}=O_{2,mix}-O_{2,E}=k_{O}(O_{2,O}-$ $O_{2,E})=0.5\cdot AMP\cdot A\cdot(1-B)(O_{2,O}-$ $O_{2,E})/V_{E}$

**River Flushing (*F_I_*)**

The rate of change of dissolved oxygen in the estuary due to river inflows is calculated as

(6) $F_{I}=k_{I}\cdot(O_{2,I}-$ $O_{2,E})=(O_{2,I}-$ $O_{2,E})\cdot V_{I}/V_{E}$

**Sediment oxygen demand (*SOD*)**

Sediment oxygen demand (*SOD*) is the rate at which dissolved oxygen is removed from the overlaying water column by biochemical processes in the sediment. The oxygen uptake by organic matter mineralization within the sediment is estimated as

(7) $SOD=a\cdot\left( \theta_{sed}^{OM} \right)^{T-20}\cdot A/V_{E}$.

**Atmospheric exchange (*Atm*)**

Oxygen can slowly diffuse across the water surface from the atmosphere. Net oxygen flux at the surface, i.e. the atmospheric exchange (*Atm*), is estimated using the algorithm presented by Riley & Skirrow (1974):

(8) $Atm= k_{e}\cdot(O_{2,S}-\gamma\cdot pO_{2,w})\cdot A/V_{E}$, where $k_{e}$ is proportional to *S_c_*^1/2^ and varies due to wind speed and water solubility (Ho *et al*., 2011) experienced within the estuary and $\gamma$, the O_2_ solubility (mg L^-1^ atm^-1^) was calculated from estuary water temperature and salinity (Weiss, 1974) as

(9) $\gamma=1.42763\cdot e^{(B1-B2+B3)}$ , where

(10) *B1* = −173.4292 + 249.6339 $\cdot$ 100.0/(*T* + 273) + 143.3483 $\cdot$ log[(*T* + 273)/100.0] , and

(11) *B2* = −21.8492 $\cdot$ (*T*+273)/100.0

(12) *B3* = *S_E_* $\cdot$ (−0.033096 + 0.014259 $\cdot$ (*T* + 273)/100.0 − 0.0017 $\cdot$ ( (*T* + 273)/100.0) ^2^)

(13) $S_{c}=(0.9+\frac{S_{E}}{35})\cdot(2073.1-125.62\cdot T+3.6276\cdot T^{2}-0.043219\cdot T^{3})$

(14) $k_{e}=0.06+0.266\cdot U_{10}^{2}/\sqrt{{S_{c}}/{660}}$ . Note that in an estuarine environment the rate of inflow will determine the extent to which the salt-wedge intrudes and subsequently the stratification patterns and DO concentrations through the water column. A regression of long-term monitoring DO data in the Swan River Estuary revealed that the ratio of depth-averaged DO concentration to the surface DO concentration decreases with the decline of inflow rate (Department of Water and Environmental Regulation, 2008-15). The surface DO concentration of this estuary is therefore calculated as

(15) $O_{2,S}=O_{2,mix}/R_{i}$ , where $R_{i}$ is the ratio of the depth-averaged DO to the DO at the surface water and where, in the Swan River Estuary, $R_{i}$ is related to inflow *V_I_* (Figure S1, a) using the fitted regression equation

(16) $R_{i}=0.028V_{IN}^{0.642}+0.655$, where inflow is converted to m^3^ s^-1^. In the current study, the DO of the bottom water is of more interest as the bottom water is the habitat for demersal fish. The ratio of DO at the bottom to the depth-averaged DO (*R_ib_*) also shows a decline with inflow *V_I_* (Figure S1) as less inflow enhanced the water stratification.

**Phytoplankton productivity and respiration (P/R)**

The photosynthetic production of oxygen is estimated as the daily net photosynthetic production by pelagic plankton (*P*) minus respiration (*R*). The net photosynthesis is estimated using Vollenweider type approximation (Reynolds, 1997), which is a function of photon density, and maximum production of *O_2_*.

(17) $P=c_{NP}\cdot N\cdot P_{max}\cdot\phi\cdot\frac{ln(0.63\cdot\frac{I_{o,max}}{I_{k}})}{{1.33}^{(e_{w}+e_{p}+N\cdot e_{s})}}$ .

Compared to estimates of photosynthetic production, information on respiratory oxygen uptake suggests a smaller but constant rate. Reynolds (1993) suggests that respiration may be estimated as,

(18) $R=24\cdot N\cdot R_{s}$.

## 1.2 Stochastic approach for assessing possible DO levels

A stochastic approach is used to illustrate the likelihood of oxygen concentrations throughout the water column under varying inflow rates. The approach uses a Monte Carlo simulation technique to perform repetitive calculations of dissolved oxygen (DO) concentration while randomly changing key parameters within scientifically-defensible ranges for each process described in Section 1.1. The results from the stochastic approach provide a quantitative understanding of the low oxygen risk possibilities in response to the inflow rates.

The range of values of the key parameters applied in the stochastic model for the four studied estuaries are provided in Table S2. For each parameter and boundary forcing variable, a set of five random values was generated using a Matlab function for pseudo-random number generation (normrnd.m), which were chosen from a normal distribution with known/assumed mean (μ) and standard deviation (σ). This resulted in a population of 5^9^ parameter sets, from which a sample of 1 million parameter sets was then chosen randomly. The deterministic oxygen model was run repetitively 1 million times, each time using a different set of parameters. The simulation results inherited a stochastic component that forms a basis for the likelihood assessment of oxygen level in the estuarine water. Table S2 also includes parameter estimates for estuary area, volume and discharge.

**1.3 Model validation against field data in Swan River Estuary.**

To evaluate the model performance, long-term (2008-15, with weekly sampling frequency) DO monitoring data at 8 sites of Swan River Estuary (NAR, NIL, STJ, MAY, RON, KIN, SUC, MSB) were obtained from the database of the Western Australian Department of Water (2017), processed and results then compared with the steady state DO predicted by the model.

Both field and modelled data show a decline in the DO concentration in response to the drop of inflow rate (Figure S2). High inflow rate (>5 m^3^ s^-1^) is more often seen in the austral winter (April – September, wet months), while in summer (October – March, dry months) the inflow rate is typically <5 m^3^ s^-1^. The mean predicted DO concentration in winter drops from 8.6 mg L^-1^ to 5.2 mg L^-1^ in association with the decline in inflow rate from 40 m^3^ s^-1^ to zero. The rate of decline of predicted DO concentration in summer is slower than that in winter, possibly due to higher phytoplankton biomass in summer that produces oxygen. The model predictions reveal a declining nonlinear relationship between the average DO concentration and inflow rate, which well match the declining trend revealed from the field data. The maximum absolute errors between the predicted mean DO and best-fit field data are 0.54 mg O_2_ L^-1^ and 0.25 mg O_2_ L^-1^ in winter and summer, respectively. These indicate that the model, at least to some extent, broadly reflects the observed DO variations associated with the changes in inflow rate.

**References**

Adiyanti S, Hipsey MR (2015) Oxygen Mass Balance Model for Assessing De-Oxygenation Risks. Report prepared for: BMT Oceanica and WA Department of Transport.

Australian Bureau of Meteorology (2017) Climate Data Online. Location: Perth Airport. http://www.bom.gov.au/climate/data/. Accessed 15/4/2017.

Department of Water and Environmental Regulation (2008-15). Quarterly Swan Canning estuarine data reports. Perth, WA, Australia.

Ho DT, Schlosser P, Orton PM (2011) On factors controlling air-water gas exchange in a large tidal river. *Estuaries and Coasts*, 34, 1103-1116.

Norlem M, Paraska D, Hipsey MR (2013) Sediment-water oxygen and nutrient fluxes in a hypoxic estuary. In: Piantadosi J, Anderssen RS, Boland J (Eds.) MODSIM2013, 20th International Congress on Modelling and Simulation.

Reynolds CS (1993) The Ecology of Freshwater Phytoplankton. Cambridge University Press, Cambridge, 384 pp.

Reynolds CS (1997) Vegetation Processes in the Pelagic: A model for ecosystem theory. Excellence in ecology, Vol 9, Ecology Institute, Oldendroft, 371 pp.

Riley JP, Skirrow G (1974) Chemical Oceanography. Academic Press, London.

Weiss RF (1974) Carbon dioxide in water and seawater: the solubility of a non-ideal gas. *Marine Chemistry* 2, 203-215.

Western Australian Department of Water (2017) River Monitoring Stations. Government of Western Australia. http://kumina.water.wa.gov.au/waterinformation/telem/stage.cfm. Accessed 01.07.17.

**List of figures**

**Figure S1:** Ratio of dissolved oxygen (DO) concentrations at the bottom to average DO concentration throughout the water column under varying inflow rates (m^3^s^-1^) in the Swan River Estuary. represents average ratios calculated from concentrations measured at eight sites in the Swan River Estuary. Fitted line $R_{ib}=0.488V_{IN}^{0.147}+0.056.$

**Figure S2.** Relationship between freshwater discharge and mean oxygen concentrations (±1 SD) throughout the water column of the Swan River Estuary under winter and summer conditions predicted by the oxygen mass balanced model. represents average oxygen concentrations measured throughout the water column at eight sites in that estuary.

**Tables**

**Table S1.** Details of model variables and parameters.

| **Symbol** | **Description** | **Values** | **Units** | **References** |
| --- | --- | --- | --- | --- |
| $V_{T}$ | Tidal prism | Calculated | m^3^ /day |  |
| $V_{I}$ | River inflow rate | Assumed based on historical record | m^3^ /day |  |
| $V_{E}$ | Estuary volume | Table S2 | m^3^ |  |
| *A* | Estuary surface area | Table S2 | m^2^ |  |
| *T* | Water temperature | Table S2 | degree C | DWER (2008-15) |
| $S_{E}$ | Estuary salinity | Table S2 |  | DWER (2008-15) |
| $O_{2,E}$ | Estuary oxygen concentration | Calculated | mgL^-1^ |  |
| $O_{2,S}$ | Estuary surface oxygen concentration | Calculated | mgL^-1^ |  |
| $O_{2,O}$ | Oxygen concentration of ocean | Table S2 | mgL^-1^ | Ocean Atlas data |
| $O_{2,I}$ | Oxygen concentration of river | Table S2 | mgL^-1^ | Assumed saturated |
| *AMP* | Tidal Amplitude | Table S2 | m |  |
| *B* | Bar closure fraction | Table S2 |  |  |
| $U_{10}$ | Wind speed at 10 m height | Table S2 | m s^-1^ | BoM (2017) |
| $pO_{2,w}$ | Atmospheric partial pressure of oxygen at sea level | 1 | atm |  |
| *F_O_* | Oxygen input by ocean flushing | Calculated | mgL^-1^day^-1^ |  |
| *F_I_* | Oxygen input by river inflows | Calculated | mgL^-1^day^-1^ |  |
| *P* | Phytoplankton production | Calculated | mgL^-1^day^-1^ |  |
| *R* | Community respiration | Calculated | mgL^-1^day^-1^ |  |
| *SOD* | Sediment oxygen demand | Calculated | mgL^-1^day^-1^ |  |
| $a$ | Sediment oxygen demand rate | Assumed based on previous application | g O_2_ m^-2^day^-1^ | Norlem *et al.* (2013); Adiyanti & Hipsey (2015) |
| $\theta_{sed}^{OM}$ | Temperature dependence on mineralization rate in sediment | 1.08 (Assumed based on previous application) |  | Adiyanti & Hipsey (2015) |
| *Atm* | Oxygen input by atmospheric exchange | Calculated | mgL^-1^day^-1^ |  |
| $k_{O}$ | Exchange coefficient | Calculated |  |  |
| $k_{I}$ | River inflow exchange coefficient | Calculated |  |  |
| $k_{e}$ | Air-water exchange coefficient | Calculated | cm h^-1^ | Ho *et al*. (2011) |
| S_c_ | Schmidt number | Calculated |  |  |
| $R_{i}$ | the ratio of surface oxygen concentration to average oxygen concentration | Calculated |  |  |
| $\gamma$ | Oxygen solubility | Calculated | mgL^-1^atm^-1^ |  |
| $c_{NP}$ | Net production efficiency coefficient | 0.67 - 0.83 |  | Reynolds (1997) |
| ***N*** | chlorophyll-*a* concentration | Calculated | μgL^-1^ | DWER (2008-15) |
| $P_{max}$ | Light saturated rate of photosynthesis | 2.28 | mg O_2_ mgChl-*a*^-1^ h^-1^ | Reynolds (1993, 1997) |
| ∅ | Daylight time | 11 | h day^-1^ | BoM (2017) |
| $I_{o,max}$ | Daily average photosynthetic available radiation | 800 | μE m^-2^s^-1^ | Reynolds (1997) |
| $I_{k}$ | Photon flux density at half saturation of photosynthesis | 24 | μEm^-2^s^-1^ | Reynolds (1997) |
| $e_{w}$ | Natural logarithmic constant | 0.095 | m^-1^ | Reynolds, (1997) |
| $e_{p}$ | Natural logarithmic constant | 0.327 | m^-1^ | Reynolds, (1997) |
| $e_{s}$ | Natural logarithmic constant | 0.0158 | m^-1^ | Reynolds, (1997) |
| $R_{s}$ | Specific rates of respiration | 2 | mg O_2_ mgChl-*a*^-1^ h^-1^ | Reynolds (1993,1997) |

DWER (Department of Water and Environmental Regulation)

BoM (Australian Bureau of Meteorology)

**Table S2.** Range of values of key parameters applied in stochastic model (mean$\pm$sd)

| Symbol | Description | Swan River Estuary | | | Moore River Estuary | | Walpole-Nornalup Inlet | | Wellstead/Beaufort Inlet | |
| --- | --- | --- | --- | --- | --- | --- | --- | --- | --- | --- |
|  |  | Winter | Summer | | Winter | Summer | Winter | Summer | Winter | Summer |
| *AMP* | Tidal Amplitude (m) | 0.16±0.05 | 0.13±0.04 | | 0.8±0.2 | 0.7±0.2 | 0.16±0.05 | 0.13±0.04 | 0.16±0.05 | 0.13±0.04 |
| *B* | Bar closure fraction (dimensionless) | 0.0±0.0 | 0.0±0.0 | | 0.6±0.2 | 1.0±0.0 | 0.0±0.0 | 0.0±0.0 | 0.7±0.1 | 0.8±0.1 |
| *T* | Water temperature (degrees) | 16.0$\pm$4.0 | 21.0$\pm$4.0 | | 20$\pm$3.0 | 25.0$\pm$4.0 | 12.0$\pm$2.0 | 18.0$\pm$3.0 | 13.0$\pm$2.0 | 19.0$\pm$3.0 |
| *S_E_* | Estuary salinity | 10.0$\pm$4.0 | 20.0$\pm$5.0 | | 4.0$\pm$1.0 | 6.0$\pm$1.0 | 11.0$\pm$2.0 | 22.0$\pm$3.0 | 11.0$\pm$2.0 | 38.0$\pm$6.0 |
| ***N*** | Chlorophyll-a concentration (µg L^-1^) | 6.0$\pm$4.0 | 20.0$\pm1$0.0 | | 6.0$\pm$4.0 | 15.0$\pm1$0.0 | 6.0$\pm$4.0 | 15.0$\pm1$0.0 | 6.0$\pm$4.0 | 15.0$\pm1$0.0 |
| $O_{2,oc}$ | Ocean oxygen concentration (mg L^-1^) | 6.5$\pm$2.0 | 7.0$\pm$2.0 | | 7.0$\pm$2.0 | 5.9$\pm$2.0 | 7.6$\pm$2.0 | 6.6$\pm$2.0 | 7.5$\pm$2.0 | 6.5$\pm$2.0 |
| $O_{2,I}$ | River O_2_ concentration (mg L^-1^) | 9.0$\pm$2.0 | 7.6$\pm$2.0 | | 8.8$\pm$1.0 | 7.4$\pm$2.0 | 9.7$\pm$2.0 | 8.4$\pm$2.0 | 10.0$\pm$2.0 | 8.3$\pm$2.0 |
| $a$ | Sediment oxygen demand rate (g O_2_ m^-2^ day^-1^) | 1.4$\pm$0.3 | 1.6$\pm$0.6 | | 1.4$\pm$0.3 | 1.8$\pm$0.6 | 1.4$\pm$0.3 | 1.8$\pm$0.6 | 1.4$\pm$0.3 | 1.8$\pm$0.6 |
| $U_{10}$ | Average wind speed (m s^-1^) | 3.3$\pm$2.0 | 4.4$\pm$2.0 | | 3.0$\pm$1.0 | 4.7$\pm$1.5 | 4.0$\pm$1.5 | 4.6$\pm$1.5 | 3.0$\pm$1.0 | 3.5$\pm$1.0 |
| *A* | Estuary area (m^2^) | 3,240,000 | 3,240,000 | 400,000 | | 400,000 | 900,000 | 900,000 | 1,440,000 | 1,440,000 |
| $V_{E}$ | Estuary volume (m^3^) | 9,720,000 | 9,720,000 | 800,000 | | 800,000 | 2,700,000 | 2,700,000 | 2,880,000 | 2,880,000 |
| $V_{I}$ | Volume of inflow waters (m^3^/day) | 0 – 4,000,000 | 0 – 4,000,000 | 300,000 | | 300,000 | 1,500,000 | 1,500,000 | 400,000 | 400,000 |
